# Multi-morbidity and its association with common cancer diagnoses: a UK Biobank prospective study

**DOI:** 10.1186/s12889-023-16202-9

**Published:** 2023-07-06

**Authors:** Megan C. Conroy, Gillian K. Reeves, Naomi E. Allen

**Affiliations:** Nuffield Department of Population Health, University of Oxford, Oxford, OX3 7LF UK

**Keywords:** UK Biobank, Cancer risk, Multi-morbidity

## Abstract

**Background:**

Whilst multi-morbidity is known to be a concern in people with cancer, very little is known about the risk of cancer in multi-morbid patients. This study aims to investigate the risk of being diagnosed with lung, colorectal, breast and prostate cancer associated with multi-morbidity.

**Methods:**

We investigated the association between multi-morbidity and subsequent risk of cancer diagnosis in UK Biobank. Cox models were used to estimate the relative risks of each cancer of interest in multi-morbid participants, using the Cambridge Multimorbidity Score. The extent to which reverse causation, residual confounding and ascertainment bias may have impacted on the findings was robustly investigated.

**Results:**

Of the 436,990 participants included in the study who were cancer-free at baseline, 21.6% (99,965) were multi-morbid (≥ 2 diseases). Over a median follow-up time of 10.9 [IQR 10.0–11.7] years, 9,019 prostate, 7,994 breast, 5,241 colorectal, and 3,591 lung cancers were diagnosed. After exclusion of the first year of follow-up, there was no clear association between multi-morbidity and risk of colorectal, prostate or breast cancer diagnosis. Those with ≥ 4 diseases at recruitment had double the risk of a subsequent lung cancer diagnosis compared to those with no diseases (HR 2.00 [95% CI 1.70–2.35] *p* for trend < 0.001). These findings were robust to sensitivity analyses aimed at reducing the impact of reverse causation, residual confounding from known cancer risk factors and ascertainment bias.

**Conclusions:**

Individuals with multi-morbidity are at an increased risk of lung cancer diagnosis. While this association did not appear to be due to common sources of bias in observational studies, further research is needed to understand what underlies this association.

**Supplementary Information:**

The online version contains supplementary material available at 10.1186/s12889-023-16202-9.

## Background

As the population ages, the incidence of both cancer and multi-morbidity (the coexistence of two or more chronic diseases) is increasing. Studies have shown that the majority of cancer patients have at least one other disease at cancer diagnosis, with 50%—90% being multi-morbid [1–4]. Multi-morbidity is associated with reduced cancer screening behaviour [5], delayed cancer diagnosis [6], treatment selection [7], poorer survival [8] and quality of life in patients with cancer [9]. However, very little research has been undertaken to investigate the relationship between multi-morbidity and the risk of being diagnosed with cancer. It is important to understand if multi-morbidity affects the risk of cancer diagnosis, as this will assist primary-care practitioners in supporting multi-morbid patients to better understand their risk and encourage cancer screening and monitoring for cancer symptoms, where appropriate [10]. Understanding this relationship will also support future health resource planning.

Multi-morbidity could impact cancer diagnosis through a number of pathways. Increased health care utilisation of multi-morbid patients [11] could result in an apparent increased risk if more frequent use of health care provides more opportunities to diagnose cancer. Conversely, multi-morbid patients may be less likely to attend cancer screening programmes [5], or may be more likely to have their cancer symptoms attributed to previously diagnosed diseases [6], and hence be less likely to be diagnosed with cancer, or diagnosed at a later date [10]. It is possible that patients are more likely to be diagnosed with disease as a consequence of diagnostic tests for cancer, and so associations between multi-morbidity and cancer may be due, at least in part, to a form of reverse causation bias [12]. It is also possible that multi-morbidity may be associated with cancer risk due to shared risk factors (i.e. confounding), for example obesity [13] and smoking [14]. On the other hand, multi-morbidity could be causally associated with cancer risk through biological pathways such as increased inflammation [15]. Given the number of potential routes that multi-morbidity could impact cancer diagnosis, it is important to robustly investigate how potential biases may impact on any observed association.

Prior research of the association between multi-morbidity and subsequent cancer diagnosis is limited. One previous study reported an increased risk of bladder and cervical cancer, but not other cancers, in multi-morbid individuals [3]. Others have found no association with colorectal cancer [16] or breast cancer [17].

The Cambridge Multi-morbidity Score (CMS) [18] was developed as a simple tool to help healthcare planners respond to the needs of patients with multiple health conditions, and to aid research into multi-morbidity [18]. The CMS was selected for this study as it was developed using a U.K. general population dataset (CPRD), and hence contains health conditions that are of high importance within the U.K. health service and to individual patients. As such, they should be well captured in both medical (i.e. primary care) and self-reported health data [18].

The current study uses UK Biobank (UKB), a large prospective UK cohort, to investigate the association between multi-morbidity and the four most common cancers in the UK (colorectal, breast, lung and prostate) and to determine the extent to which any association might be explained by ascertainment bias, shared risk factors or reverse causation bias.

## Methods

### Participants

UKB is a large, prospective cohort study in which ~ 500,000 volunteers underwent a comprehensive baseline assessment between 2006–2010, providing in-depth data on socio-demographics, lifestyle and health, and a range of physical measures. Participants consented to linkage to their electronic health records for longitudinal follow-up of health outcomes. Full details of the UKB cohort are given elsewhere [19].

Linkages have been undertaken to national cancer (data available from 1979 onwards) and death (2006 onwards) registries, hospital inpatient data (1997 onwards) and, for ~ 45% of the cohort, primary-care records (1938 – 2017).

UKB received ethics approval from the National Information Governance Board for Health and Social Care and the National Health Service North West Multicentre Research Ethics Committee (21/NW/0157).

In the current analysis, only UKB participants with no evidence of pre-existing cancer were included in the study.

### Multi-morbidity ascertainment

The CMS [18] is a weighted multi-morbidity score, which is formed of 37 different conditions selected for their substantial impact on patients, based on expert opinion [18]. For the purposes of this study, four of these conditions were excluded from the assessment of multi-morbidity, including cancer (since this was the outcome of interest), vision/ hearing loss and painful conditions (as not well defined in UKB), and learning difficulties (owing to its low prevalence due to the consenting procedures for UKB). The remaining 33 conditions were used to determine the CMS in this study.

At recruitment, all UKB participants were asked via a touch-screen questionnaire *"Has a doctor ever told you that you have had any serious medical conditions or disabilities?”* and underwent a detailed nurse-led interview, where a full medical history was collected. This self-reported health data collected at recruitment was used to determine the CMS as the exposure for the main analysis. In sensitivity analyses aimed at assessing potential ascertainment bias, the main analyses were repeated used linked primary care data (in the subset of ~ 45% participants for whom these data were available) to identify health conditions included in the CMS that were recorded prior to recruitment (that is, an “ever diagnosed” definition was used).

Multi-morbidity at recruitment was defined in multiple ways, as follows: i) ≥ 2 diseases, ii) weighted CMS (using published weights [18]) categorised into fourths (with those with no diseases as a separate category), and iii) a categorical variable representing number of conditions (0, 1, 2, 3 and ≥ 4). This latter measure of disease count was used as the primary exposure of interest owing to its ease of interpretation.

Code lists for disease definitions are available at 10.5281/zenodo.7334984.

### Cancer ascertainment

The cancer registry data was used to identify cases during the follow-up period. Participants with a cancer diagnosis prior to recruitment (excluding non-melanoma skin cancer) were considered prevalent cases and excluded from the analysis. The first incident cancer diagnosis (excluding non-melanoma skin cancer) was identified for remaining participants. The four most commonly diagnosed cancers in UKB were selected as outcomes of interest because of their high public health impact and large numbers of incident cases. Prostate cancer was defined as ICD-10 code C61, breast cancer as C50, lung cancer as C33-34 and colorectal cancer as C18-20.

### Co-variates

Date of birth was estimated from month and year of birth. Townsend deprivation score (an area-based score denoting socio-economic deprivation of a participant’s residence at recruitment) was categorised into fifths. Region was defined as London; South East; South West; East Midlands; West Midlands; North East; North West; Yorkshire and the Humber; Scotland East; Scotland West; Wales. Ethnicity was characterised as white and non-white.

Body mass index (BMI) was categorised as < 25; 25–30; ≥ 30 kg/m^2^. Smoking was categorised as never; former; < 10 cigarettes daily; ≥ 10 cigarettes daily; current smoker with unknown number of cigarettes daily. Alcohol consumption was categorised as never; special occasions only; 1–3 times per month; 1–2 times per week; 3–4 times per week; daily or almost daily. Menopausal hormonal therapy (MHT) use was categorised as never or ever users (women only).

Self-reported screening behaviour was categorised as never or ever attended breast or bowel cancer screening and prostate specific antigen (PSA) testing. To further assess the extent to which overall health care utilisation might influence the association between multi-morbidity and cancer diagnosis, the following data were extracted from the primary-care records at recruitment: rapid referral for cancer diagnostics (yes or no), number of consultations (i.e. number of times a participant had attended their primary-care clinician; categorised as fifths), number of spirometry measurements, number of blood pressure measurements, and number of PSA tests (each categorised as 1, 2, 3, 4, ≥ 5).

### Analyses

Individuals contributed person-years to the analysis from their date of recruitment (2006–2010) until the earliest of: date of first cancer diagnosis (excluding non-melanoma skin cancer), loss to follow up (e.g. withdrawal from study or leaving the U.K.), death, or end of follow up. End of follow up was defined as the date for which cancer registry data were considered complete (England and Wales 29^th^ February 2020; Scotland 31^st^ January 2021).

Distribution of multi-morbidity measures according to various characteristics were calculated and summarised as median and interquartile range (IQR), mean and standard deviation (SD) or proportions.

Cox proportional hazards models, with attained age as the underlying time variable, were used to investigate the association between the multi-morbidity metrics at recruitment and subsequent cancer diagnosis during the follow-up period. Initial investigations compared these associations across follow-up intervals (< 1, 1–5, ≥ 5 years) and heterogeneity was assessed using likelihood ratio (LR) tests. All subsequent analyses focused on associations of multi-morbidity with cancer risk in the period 1 or more years after the recording of morbidity data (i.e. excluding the first year of follow-up) to minimise the impact of reverse causation.

Initial models were adjusted for year of birth, region, socio-economic status and ethnicity. Proportionality was checked by examining Schoenfeld’s residuals. To investigate the possibility of residual confounding (i.e. to determine if any association might be partly explained by the presence of shared risk factors), the models were further adjusted for BMI, alcohol consumption, smoking and MHT use. The change in LRχ^2^statistics associated with multi-morbidity were estimated before and after adjustment for these confounders [20]. For the purposes of this study, a reduction in the LRχ^2^ of more than two-thirds after adjustment for all potential confounders was taken to indicate that the adjusted association could plausibly be due to residual confounding (caused by imperfect adjustment for the confounder(s) under study). Given the particularly strong association between smoking and lung cancer and other conditions included in the CMS, analyses of multi-morbidity with lung cancer was also restricted to never smokers to further investigate the possibility of residual confounding. To assess if ascertainment bias might have influenced the association, breast, colorectal, and prostate cancer models were adjusted for self-reported routine screening behaviour.

In participants for whom primary care information were available the mean number of consultations, spirometry measurements, blood pressure measurements and PSA tests (men only) were calculated stratified by the number of diseases, in those without a cancer diagnosis. The association of multi-morbidity with cancer diagnosis among those with primary-care data was then further adjusted for the number of consultations (as a proxy of health care utilisation) – and for prostate cancer, the number of PSA tests – to determine the extent to which healthcare usage might explain the associations.

Where significant associations of multi-morbidity with cancer diagnosis were identified, associations between individual diseases within the CMS and cancer risk were investigated using the same methodology as for the multi-morbidity metrics. Multiple testing was adjusted for using the false discovery rate [21].

## Results

Of the 502,411 participants in UKB, 34,161 (6.9%) had a prevalent cancer and were excluded from analysis. Over a median follow up time of 10.9 years (IQR 10.0 – 11.7)) 9,019 prostate, 7,994 breast, 5,241 colorectal, and 3,591 lung cancers were identified.

The most common self-reported diseases within the CMS were hypertension (26.5%), asthma (11.6%), and thyroid disease (5.7%) (Supplementary Table 1). Just over a fifth of participants were multi-morbid (≥ 2 diseases: 99,965; 21.6%), with 45.8% (214,229) self-reporting none of the 33 conditions included in the CMS (Table 1).

**Table 1:** Distribution of co-variates by self-reported number of diseases, restricted to those with at least 12 months follow up

Co-variate	Number of Diseases				
	0	1	2	3	≥ 4
**Age (median (IQR))**	55 (48—61)	58 (51—63)	60 (53—65)	61 (55—65)	61 (55—65)
**Sex** (*n* = 463,990)					
Female	114,486 (46.34%)	81,459 (32.97%)	34,488 (13.96%)	11,639 (4.71%)	4979 (2.02%)
**Ethnicity** (*n* = 460,923)					
White	199,338 (45.8%)	143,097 (32.9%)	62,400 (14.3%)	21,238 (4.9%)	9059 (2.1%)
**SES (median (IQR))**	-2.3 (-3.7—0.2)	-2.2 (-3.7—0.5)	-1.97 (-3.5—1.0)	-1.5 (-3.3—1.7)	-0.7 (-3.0—2.7)
**BMI (median (IQR))**	25.9 (23.6—28.7)	27.0 (24.4—30.2)	28.0 (25.2—31.6)	28.9 (25.8—32.8)	30.2 (26.7—34.5)
**Smoking** (*n* = 462,344)					
Current	21,981 (45.1%)	15,382 (31.6%)	7160 (14.7%)	2792 (5.7%)	1437 (2.9%)
**Alcohol** (*n* = 462,127)					
Daily	43,979 (46.8%)	31,708 (33.7%)	12,853 (13.7%)	3948 (4.2%)	1477 (1.6%)
**MHT** (*n* = 247,051)					
Ever	35,295 (38.0%)	32,636 (35.2%)	15,987 (17.2%)	6045 (6.5%)	2883 (3.1%)

Multi-morbid participants were older, lived in more socio-economically deprived areas, had a higher BMI, were more likely to smoke, less likely to drink alcohol and, among women, more likely to have ever used MHT (Table 1).

To assess the possibility of reverse causation, an initial time-stratified analysis was undertaken, which showed an increased risk of number of diseases with colorectal, breast and lung cancer in the first 12 months of follow-up that was either partially or completely attenuated with longer follow up (Supplementary Fig. 1; Supplementary Tables 2 and 3).

**Fig. 1:**
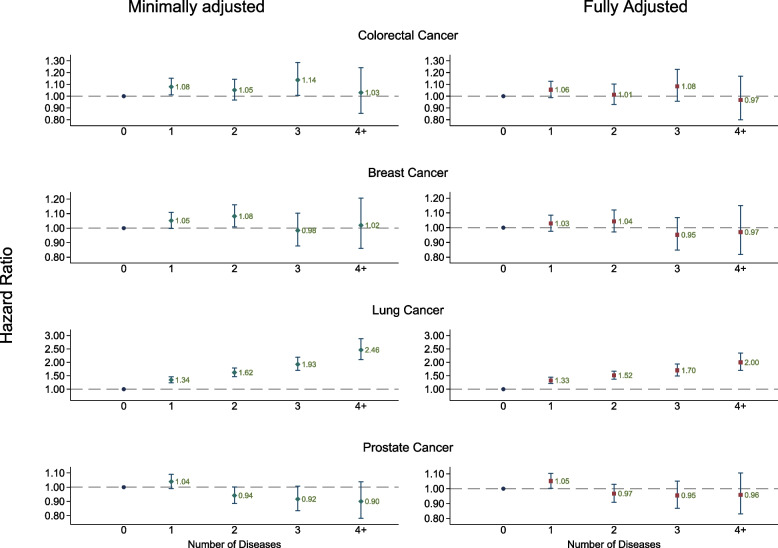
Association between disease count and risk of cancer diagnosis. Diamond: Adjusted for sex, region, year of recruitment, ethnicity year of birth and townsend score, age as underlying time variable Square: Further adjusted for BMI, smoking, alcohol consumption and HRT use (except Prostate cancer)

In subsequent analyses, which examined the relationship between multi-morbidity at baseline and risk of each type of cancer only in the follow-up period 1 or more years after baseline (in those who were still alive and at risk of a first cancer at this point),, there was no association between multi-morbidity and colorectal, breast or prostate cancer (Fig. 1; Supplementary Table 4). Additional adjustment for self-reported screening behaviour (used as a proxy for healthcare seeking behaviour) did not affect the results (data not shown).

Participants with ≥ 4 diseases had a 2.5-fold increased risk of lung cancer compared to those with no diseases, which was only partially attenuated after further adjustment for BMI, alcohol consumption, smoking and MHT use (HR 2.00 [95% CI 1.70 – 2.35] *p* for trend < 0.001) (Fig. 1; Supplementary Table 4). The LRχ^2^ between the minimally and fully adjusted models was reduced by 37%, indicating that residual confounding is unlikely to fully explain the association identified (Supplementary Table 5). The analysis restricted to the 255,850 participants that reported having never smoked (of which there were 479 incident lung cancers) showed a similar trend, with those with ≥ 4 diseases having almost double the risk of lung cancer compared to those with no diseases (1.97 [11.8 – 3.27] *p* for trend 0.03; Table 2).

**Table 2:** Sensitivity analyses: association of multi-morbidity with risk of diagnosis of site-specific cancers based on primary care and self-report (in those with primary care data), and in never smokers (for lung cancer only – self-reported data)

**Sensitivity analyses**				**Colorectal Cancer**		**Breast Cancer**		**Lung Cancer**		**Prostate Cancer**	
				HR (95% CI)	*P*^1^	HR (95% CI)	*P*^1^	HR (95% CI)	*P*^1^	HR (95% CI)	*P*^1^
**Never smokers**^**a**^		Number of diseases	0					Reference	0.03		
			1					1.17 (0.95 – 1.44)			
			2					1.27 (0.97 – 1.66)			
			3					1.20 (0.79 – 1.83)			
			≥ 4					1.97 (1.18 – 3.27)			
**Restricted to subset with GP data**	GP data^b^	Number of diseases	0	Reference	0.17	Reference	0.07	Reference	< 0.001	Reference	< 0.001
			1	0.97 (0.86—1.09)		0.97 (0.88—1.06)		1.18 (1.02—1.37)		0.96 (0.88—1.04)	
			2	1.06 (0.92—1.22)		0.95 (0.85—1.06)		1.23 (1.04 -1.44)		0.96 (0.87—1.06)	
			3	0.95 (0.79—1.14)		0.98 (0.86—1.13)		1.28 (1.06—1.55)		0.86 (0.76 – 0.98)	
			≥ 4	1.18 (0.97—1.42)		0.88 (0.75—1.03)		1.67 (1.39—2.01)		0.81 (0.70—0.93)	
	SR data^b^	Number of diseases	0	Reference	0.80	Reference	0.09	Reference	< 0.001	Reference	0.06
			1	1.03 (0.93—1.13)		0.98 (0.91—1.06)		1.31 (1.15—1.49)		1.01 (0.94—1.09)	
			2	0.97 (0.85—1.11)		1.00 (0.90—1.12)		1.40 (1.20—1.63)		0.90 (0.81—1.00)	
			3	1.19 (0.99—1.43)		0.79 (0.66—0.95)		1.80 (1.49—2.18)		1.00 (0.87—1.15)	
			≥ 4	0.75 (0.54—1.03)		0.85 (0.65—1.10)		1.82 (1.42—2.33)		0.77 (0.61—0.97)	

*HR* Hazard ratio, *CI* Confidence interval, *SR* Self-report

^a^Adjusted for sex, year of birth, year of recruitment, townsend score, ethnicity, region, BMI, alcohol consumption and MHT use

^b^Adjusted for sex (colorectal and lung cancer only), year of birth, year of recruitment, townsend score, ethnicity, region, BMI, alcohol consumption, smoking, MHT use, number of consultations, rapid referral and PSA tests (prostate cancer only)

^1^*p* value for trend

Associations of the other multi-morbidity indices with risk of cancer diagnosis were broadly similar. For brevity, only the findings with respect to disease count are included in the main text. Full details of results for other multi-morbidity indices are given in Supplementary Table 4.

There was little difference in the association between multi-morbidity and risk of cancer diagnosis when stratified by 1- < 5 years and ≥ 5 years follow-up, with no evidence of heterogeneity for any outcome (Supplementary Fig. 1, Supplementary Tables 2 and 3).

**Fig. 2:**
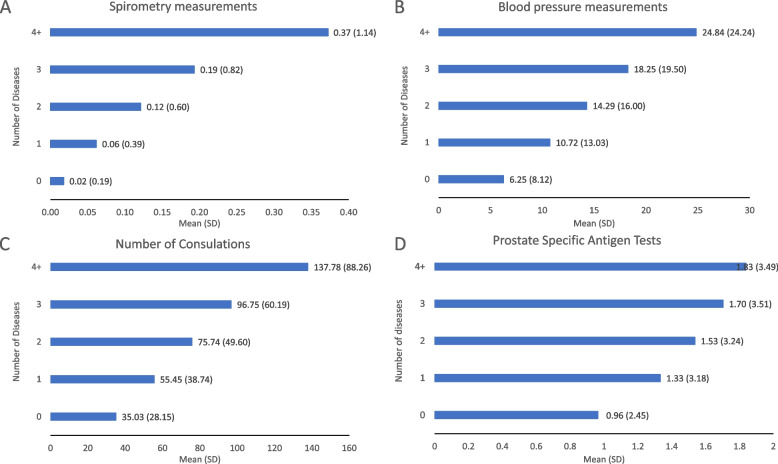
**A** mean number of spirometry measurements recorded at recruitment in primary-care records, by number of diseases and cancer diagnosed; **B** mean number of blood pressure measurements recorded at recruitment in primary-care records, by number of diseases and cancer diagnosed; **C** mean number of consultations recorded at recruitment in primary-care records, by number of diseases and cancer diagnosed; **D** mean number of prostate specific antigen tests (men only)

Overall, 203,916 (43.9%) had a primary-care record at recruitment, of which there were 4,096 incident prostate, 3,364 breast, 2,224 colorectal and 1,573 lung cancers diagnosed at least 12 months after recruitment. In contrast with self-reported conditions at recruitment, the most common diseases ascertained from primary-care data in the CMS were psoriasis and eczema (16.8%), anxiety (12.6%), and depression (14.4%). A third of participants were multi-morbid (33.2%; *n* = 67,755), while 38.0% (77,443) did not have any of the CMS diseases recorded in their primary-care record (Supplementary Table 1). The distribution of covariates by number of diseases was similar to that seen for the self-reported derived diseases (Supplementary Table 6). Given that the number of consultations, blood pressure measurements, spirometry measurements and PSA tests derived from the primary-care data (Fig. 2) were all positively correlated with increasing disease count, thus indicating that multi-morbidity could lead to increased opportunity for cancer diagnosis, analyses of multi-morbidity and risk of cancer diagnosis were further adjusted for number of consultations (owing to the significant correlation with the other measures of healthcare utilisation; pairwise correlation coefficient significance all *p* < 0.001), rapid referral for cancer diagnostic investigations and, for prostate cancer only, PSA testing. When analyses of multi-morbidity were conducted in this subset of participants with primary care data, with additional adjustment for markers of healthcare utilisation,, similar patterns of associations were observed regardless of whether multi-morbidity was based on primary care data or self-report. In particular, multi-morbidity was not associated with colorectal cancer or breast cancer but was positively associated with lung cancer (Table 2), suggesting that ascertainment bias does not fully explain this association. However, multi-morbidity was inversely associated with prostate cancer with those with ≥ 4 diseases having a 19% lower risk compared to those with no diseases (0.81 [0.70–0.93] *p* for trend < 0.001) (Table 2).

Following correction for multiple testing, self-reported alcohol problems, asthma, chronic obstructive pulmonary disease (COPD), coronary artery disease, constipation, diabetes, hypertension, inflammatory bowel disease, peptic ulcer disease, peripheral vascular disease, substance misuse and stroke or transient ischemic attack were all associated with incident lung cancer (Supplementary Table 7).

## Discussion

This large prospective study has shown that self-reported multi-morbidity, when measured as a score, count or binary variable, is associated with an increased risk of lung cancer diagnosis, but not with risk of diagnosis of colorectal, breast or prostate cancer. This association remained relatively unaffected in sensitivity analyses aimed at minimising the impact of potential reverse causation, ascertainment bias and residual confounding. The risk of lung cancer diagnosis was associated with 12 of the 33 individual conditions considered in assessment of multi-morbidity and so no specific disease appeared to underlie this association. However, a number of the diseases (e.g. asthma, COPD, diabetes, coronary artery disease, hypertension) are associated with increased inflammation [22–26], and so there may be an inflammatory (local or systemic) element to the identified association, but this needs to be investigated further.

### Interpretation of results

As it is common for a number of conditions to be diagnosed on the cancer diagnostic pathway [12], and it is also possible that patients with more diseases are more likely to be investigated for cancer, it is important to consider the effects of reverse causation on these findings (i.e. that the disease is more likely to be diagnosed because of tests being undertaken as part of the cancer diagnosis, or that an undiagnosed cancer causes the disease, which is diagnosed prior to the cancer). Indeed, there was a notably greater increase in the risk of 3 of the 4 cancers considered here in the first year of follow up, compared with later periods, suggesting this is likely. However, there were no obvious changes in cancer risk across subsequent follow-up periods, indicating that any effect of reverse causation was largely confined to the first year of follow-up.

Given the known contribution of certain modifiable lifestyle factors, such as obesity and smoking, to many of the diseases included in the CMS and some of the cancer outcomes, it is important to investigate if shared risk factors explain any observed associations. In this study, adjustment for potential shared risk factors suggests that residual confounding from these factors are unlikely to explain the associations. Further, the association between multi-morbidity and lung cancer was unaffected when limited to self-reported never-smokers, suggesting that this association is not fully explained by smoking. Although it is possible that passive smoking might have contributed to the increased risk, it is highly unlikely to explain all of the increased risk, given the magnitude of the association.

The association with lung cancer was also evident when using primary-care data to ascertain multi-morbidity, despite the differences in the distributions of diseases between the two data sources, and there was no single condition that appeared to underlie this association. Furthermore, additional adjustment for metrics of health care utilisation (to account for the possibility that multi-morbid participants may be more likely to be investigated for symptoms simply due to attending a health care provider more frequently) did not materially affect the association with lung cancer, indicating that it is unlikely to be explained by ascertainment bias. Further research on stage of lung cancer would be useful to confirm this is the case.

Whilst no association was identified between multi-morbidity and prostate cancer using the self-reported health data, an inverse association was observed when using the primary-care data. Although this could be due to ascertainment bias (particularly given the higher prevalence of PSA testing in the UKB cohort compared with the general UK population [27, 28]), the difference in mean number of PSA tests was less than 1 between those with no diseases and those with ≥ 4 diseases, so PSA testing is unlikely to fully explain the apparent reduction in prostate cancer risk. Further, the inclusion of PSA testing as a covariate did not change the risk estimate materially. Instead, it is possible that prostate cancer might be diagnosed earlier in those with fewer diseases (as those with multi-morbidity may delay getting symptoms investigated, or the symptoms may be attributed to an already diagnosed disease, such as prostate disorder), leading to an apparent lower risk in prostate cancer in those with more diseases. However, data on cancer stage is required to investigate this further.

### Previous research

Previous research into multi-morbidity as a risk factor for cancer has been limited, with most research focussing on the association with survival following cancer diagnoses [29, 30], the prevalence and/or risk of multi-morbidity in cancer patients [2, 31], or the impact of multi-morbidity on living with cancer (including treatment options) [9, 32]. A cross sectional study in the UK found that almost half of cancer patients have at least 2 morbidities (from a list of 11) at cancer diagnosis [4], which was particularly apparent for those with gallbladder, liver, lung, myeloma and renal cancer when compared to those with colorectal cancer [4]. A US cross-sectional study identified an association between multi-morbidity (defined as ≥ 2 diseases from a list of 6) and all site, bladder and cervical cancer, but no association with colon, lung, breast or prostate cancer [3]. However, different methodology (including diseases included and a lack of data on when diseases were diagnosed in relation to cancer) may explain the differences in results seen in the current study. A recent prospective study, also using UKB data, identified no association between multi-morbidity (defined as ≥ 2 diseases from a list of 43) and colorectal cancer risk [16]. Another recent UKB study identified no association between multi-morbidity, defined as disease count or as disease clusters, and breast cancer risk [17]. Both of these studies are consistent with our findings despite differences in cohort selection and the diseases included in the multi-morbidity definition. As the results seen in our study appear to be robust to reverse causation, residual confounding and ascertainment bias, it is possible there is an aetiological role for multi-morbidity in lung cancer risk. Both asthma [33] and COPD [34] have previously been identified as risk factors for lung cancer, and both of these conditions (in addition to several others) were found to be associated with an increased risk in our study, and so it is possible that these diseases are important contributors to the association. It is also possible that multi-morbidity results in a general increase in systemic inflammation [15], which could predispose to further disease.

### Strengths and limitations of the study

This study has many strengths, including the large sample, prospective analyses and use of a validated score. As far as we are aware, this is the first study to use a multi-morbidity score (the CMS [18]) to define multi-morbidity for the investigation of cancer risk. This score was originally developed using UK primary-care data, and the disease list was determined using a combination of prevalence, impact on health care usage and impact on the patient [35]. Additionally, results were compared to those generated from primary-care records for a subset of the cohort, and despite the different distribution of some of the health conditions, the association for lung cancer remained consistent. However, there are some limitations to the study. The main analyses were undertaken using self-reported data (as not all participants had primary-care data available) but whilst it is possible that individuals may not always accurately report their morbidity status, this is of less of a concern for symptomatic diseases or those that result in a lifestyle change [36], as can be seen when comparing the prevalence of diseases from the self-reported and primary-care data in this study. Secondary-care data are available in UKB, but was not used here as some of the diseases included in the CMS are not well captured in hospital in-patient records, and so could be under ascertained. The use of self-reported data meant that changes in multi-morbidity over time could not be taken into account in the analysis. Further, the use of composite scores or disease counts may not be that informative in terms of understanding which diseases or disease patterns and associations are underling any increased risk identified. Misclassification of smoking status cannot be ruled out (as it was self-reported), however adjustments for smoking did not substantially change the point estimate for lung cancer diagnosis risk, and the association was still evident in those who reported never smoking Finally, owing to the voluntary nature of the study, UKB participants are not representative of the general population [28, 37, 38]. As such, it is not appropriate to generalise prevalence estimates of the individual diseases reported here to the general population, although there is no reason to expect that the observed association of multi-morbidity with lung cancer would not be generalizable to the wider population. As an observational analysis, causality cannot be determined from these results, and there is the possibility that unmeasured confounders, or other biases, could have contributed to the associations identified.

## Conclusions, impact and further research

This study has highlighted an increased risk of lung cancer diagnosis in those with multi-morbidity, which does not appear to be explained fully by reverse causality, residual confounding or ascertainment bias. This provides evidence to clinicians that their multi-morbid patients may be at increased risk of lung cancer, and provides evidence for health resource planners given the increasing prevalence of multi-morbidity in the U.K. However, what cannot be identified from the use of a score is which diseases or combinations of diseases might explain this association. Further research is required to assess whether there are any particular groups of multi-morbid patients that are at increased risk of lung cancer, any particular pre-existing morbidities that increase the risk of lung cancer diagnosis, and to elucidate the drivers of such associations. While the reasons for the increase in lung cancer risk among multi-morbid individuals are not yet clear, clinicians, health care planners and patients should be aware of the increased risk identified.

## Supplementary Information

**Additional file 1: Supplementary Table 1.** Prevalence of diseases included in Cambridge Multimorbidity Score in UK Biobank at baseline, by data source. **Supplementary Figure 1**. Association between disease count and risk of cancer diagnosis stratified by follow up time in UK Biobank. **Supplementary Table 2.** Association between self-reported derived multi-morbidity measures and risk of colorectal and prostate cancer diagnosis by follow-up time in UK Biobank. **Supplementary Table 3**. Association between self-reported derived multi-morbidity measures and risk of breast and lung cancer diagnosis by follow-up time in UK Biobank. **Supplementary table 4**. Association between multi-morbidity measures and risk of diagnosis of colorectal, breast, lung and prostate cancer. **Supplementary Table 5.** LRχ^2^ values for minimally and fully adjusted models for each cancer. **Supplementary Table 6.** Distribution of covariates by number of diseases in the UK Biobank subset with primary care data, restricted to those with at least 12 months follow up. **Supplementary Table 7.** Association between self-reported individual diseases at recruitment and risk of diagnosis of lung cancer.

## Data Availability

Data is available to registered researchers directly from UK Biobank. Applications can be made at https://www.ukbiobank.ac.uk/enable-your-research/apply-for-access. The underlying code to generate the results in the manuscript will be returned to UK Biobank and made available to researchers at UK Biobank’s discretion. The code lists used to ascertain CMS diseases from the UK Biobank data are available at https://github.com/meganconroy/healthphenotypes

## Abbreviations

CMS: Cambridge Multimorbidity Score
UKB: UK Biobank
MHT: Menopausal hormonal therapy
PSA: Prostate specific antigen
IQR: Interquartile range
SD: Standard deviation
LR: Likelihood ratio
HR: Hazard ratio
CI: Confidence interval
COPD: Chronic obstructive pulmonary disease

## References

[1] Koné AP, Scharf D. Prevalence of multimorbidity in adults with cancer, and associated health service utilization in Ontario, Canada: a population-based retrospective cohort study. BMC Cancer. 2021;21:406. 10.1186/s12885-021-08102-1. 10.1186/s12885-021-08102-1 PMC8048167 33853565

[2] Fowler H (2020). Comorbidity prevalence among cancer patients: A population-based cohort study of four cancers. BMC Cancer.

[3] Tang F, Gates Kuliszewski M, Carrascal A, Vásquez E (2021). Physical multimorbidity and cancer prevalence in the National Health and Nutrition Examination Survey. Public Health..

[4] Koo MM (2020). The prevalence of chronic conditions in patients diagnosed with one of 29 common and rarer cancers: A cross-sectional study using primary care data. Cancer Epidemiol.

[5] Coronado GD, Nielson CM, Keast EM, Petrik AF, Suls JM. The influence of multi-morbidities on colorectal cancer screening recommendations and completion. Cancer Causes Control. 2021;1–11. 10.1007/s10552-021-01408-2. 10.1007/s10552-021-01408-2 PMC8717879 33687606

[6] Mounce LTA, Price S, Valderas JM, Hamilton W (2017). Comorbid conditions delay diagnosis of colorectal cancer: a cohort study using electronic primary care records. Br J Cancer..

[7] Sarfati D, Koczwara B, Jackson C (2016). The impact of comorbidity on cancer and its treatment. CA Cancer J Clin.

[8] Bensken WP (2022). Comparing the association between multiple chronic conditions, multimorbidity, frailty, and survival among older cancer patients. J Geriatr Oncol.

[9] Haase KR, Hall S, Sattar S, Ahmed S (2021). Living with cancer and multimorbidity: A qualitative study of self-management experiences of older adults with cancer. Eur J Oncol Nurs.

[10] Mazza D, Mitchell G (2017). Cancer, ageing, multimorbidity and primary care. Eur J Cancer Care (Engl).

[11] Soley-Bori M (2021). Impact of multimorbidity on healthcare costs and utilisation: a systematic review of the UK literature. Br J Gen Pract.

[12] Maringe C, Fowler H, Rachet B, Luque-Fernandez MA (2017). Reproducibility, reliability and validity of population-based administrative health data for the assessment of cancer non-related comorbidities. PLoS ONE.

[13] Arnold M (2016). Obesity and cancer: An update of the global impact. Cancer Epidemiol.

[14] Gandini S (2008). Tobacco smoking and cancer: A meta-analysis. Int J Cancer.

[15] Ferreira GD. et al. Physiological markers and multimorbidity. 2018;8:2235042X1880698. 10.1177/2235042X18806986 10.1177/2235042X18806986 PMC6201184 30364915

[16] Corcoran NM, Mair FS, Nicholl B, Macdonald S, Jani BD. Long-term conditions, multimorbidity and colorectal cancer risk in the UK Biobank cohort. J Multimorb Comorb. 2022;12. 10.1177/26335565221110123. 10.1177/26335565221110123 PMC9483970 36132374

[17] Mawulawoe A (2023). Multi-Morbidity and Risk of Breast Cancer among Women in the UK Biobank Cohort. Cancers..

[18] Payne RA (2020). Development and validation of the Cambridge Multimorbidity Score. CMAJ.

[19] Conroy MC (2022). UK Biobank: a globally important resource for cancer research. Br J Cancer.

[20] Floud S (2016). The role of health-related behavioural factors in accounting for inequalities in coronary heart disease risk by education and area deprivation: Prospective study of 1.2 million UK women. BMC Med..

[21] Benjamini Y, Krieger AM, Yekutieli D (2006). Adaptive linear step-up procedures that control the false discovery rate. Biometrika.

[22] Donath MY, Shoelson SE (2011). Type 2 diabetes as an inflammatory disease. Nat Rev Immunol..

[23] Murdoch JR, Lloyd CM (2010). Chronic inflammation and asthma. Mutat Res.

[24] Tuttolomondo A (2009). Atherosclerosis as an Inflammatory Disease Pharmakeftiki.

[25] Li JJ, Fang CH, Hui RT (2005). Is hypertension an inflammatory disease?. Med Hypotheses.

[26] Magnussen H, Watz H (2009). Systemic inflammation in chronic obstructive pulmonary disease and asthma: Relation with comorbidities. Proc Am Thorac Soc.

[27] Littlejohns TJ, Travis RC, Key TJ, Allen NE (2016). Lifestyle factors and prostate-specific antigen (PSA) testing in UK Biobank: implications for epidemiological research. Cancer Epidemiol.

[28] Fry A (2017). Comparison of Sociodemographic and Health-Related Characteristics of UK Biobank Participants with the General Population. Am J Epidemiol.

[29] Michalopoulou E (2021). Impact of comorbidities at diagnosis on the 10-year colorectal cancer net survival: A population-based study. Cancer Epidemiol.

[30] Salas M (2021). Use of comorbidity indices in patients with any cancer, breast cancer, and human epidermal growth factor receptor-2-positive breast cancer: A systematic review. PLoS ONE.

[31] Freisling H (2020). Lifestyle factors and risk of multimorbidity of cancer and cardiometabolic diseases: A multinational cohort study. BMC Med.

[32] Blane DN, Lewandowska M (2019). Living with cancer and multimorbidity: The role of primary care. Curr Opin Support Palliat Care.

[33] Qu YL (2017). Asthma and the risk of lung cancer: a meta-analysis. Oncotarget.

[34] Park HY (2020). Chronic obstructive pulmonary disease and lung cancer incidence in never smokers: a cohort study. Thorax.

[35] Cassell A (2018). The epidemiology of multimorbidity in primary care: A retrospective cohort study. Br J Gen Pract.

[36] Violán C (2013). Comparison of the information provided by electronic health records data and a population health survey to estimate prevalence of selected health conditions and multimorbidity. BMC Public Health.

[37] Wong SL, Shields M, Leatherdale S, Malaison E, Hammond D (2012). Assessment of validity of self-reported smoking status. Health Rep.

[38] West R, Zatonski W, Przewozniak K, Jarvis MJ (2007). Can We Trust National Smoking Prevalence Figures? Discrepancies Between Biochemically Assessed and Self-Reported Smoking Rates in Three Countries. Cancer Epidemiol Biomark Prev.

