# Supplementary material for: Multi-morbidity and its association with common cancer diagnoses: a UK Biobank prospective study
Source: BMC Public Health. 2023 Jul 6;23:1300. doi: 10.1186/s12889-023-16202-9 (PMC10326925; doi:10.1186/s12889-023-16202-9)
Supplement: Supplementary file 1 — Additional file 1: Supplementary Table 1. Prevalence of diseases included in Cambridge Multimorbidity Score in UK Biobank at baseline, by data source. Supplementary Figure 1. Association between disease count and risk of cancer diagnosis stratified by follow up time in UK Biobank. Supplementary Table 2. Association between self-reported derived multi-morbidity measures and risk of colorectal and prostate cancer diagnosis by follow-up time in UK Biobank. Supplementary Table 3. Association between self-reported derived multi-morbidity measures and risk of breast and lung cancer diagnosis by follow-up time in UK Biobank. Supplementary table 4. Association between multi-morbidity measures and risk of diagnosis of colorectal, breast, lung and prostate cancer. Supplementary Table 5. LRχ2 values for minimally and fully adjusted models for each cancer. Supplementary Table 6. Distribution of covariates by number of diseases in the UK Biobank subset with primary care data, restricted to those with at least 12 months follow up. Supplementary Table 7. Association between self-reported individual diseases at recruitment and risk of diagnosis of lung cancer. [file 12889_2023_16202_MOESM1_ESM.docx]

**Supplementary Table 1: Prevalence of diseases included in Cambridge Multimorbidity Score in UK Biobank at baseline, by data source**

| Disease | Self-report | GP |
| --- | --- | --- |
|  | N (%) | N (%) |
| Alcohol Problems | 797 (0.2%) | 2437 (1.1%) |
| Anorexia/ Bulimia | 370 (0.1%) | 603 (0.3%) |
| Anxiety | 11713 (2.4%) | 27637 (12.6%) |
| Asthma | 57768 (11.6%) | 20809 (9.5%) |
| Atrial Fibrillation | 3663 (0.7%) | 3093 (1.4%) |
| Bronchiectasis | 4700 (0.9%) | 939 (0.4%) |
| Chronic Kidney Disease | 228 (0.05%) | 9163 (4.2%) |
| Chronic Liver disease | 2051 (0.4%) | 1151 (0.5%) |
| Chronic Sinusitis | 3080 (0.6%) | 6588 (3.0%) |
| Constipation | 397 (0.1%) | 6925 (3.2%) |
| COPD | 11636 (2.3%) | 2940 (1.3%) |
| Coronary artery disease | 22578 (4.5%) | 9645 (4.4%) |
| Dementia | 292 (0.1%) | 75 (0.0%) |
| Depression | 28214 (5.7%) | 27099 (12.4%) |
| Diabetes | 25401 (5.1%) | 10550 (4.8%) |
| Diverticular disease | 5351 (1.1%) | 6123 (2.8%) |
| Psoriasis or eczema | 18347 (3.7%) | 36841 (16.8%) |
| Epilepsy | 4010 (0.8%) | 2567 (1.2%) |
| Heart Failure | 304 (0.1%) | 852 (0.4%) |
| Hypertension | 131809 (26.5%) | 26306 (12.0%) |
| Inflammatory Bowel Disease | 4190 (0.8%) | 2400 (1.1%) |
| Irritable bowel syndrome | 11383 (2.3%) | 14453 (6.6%) |
| Migraine | 14279 (2.9%) | 14109 (6.4%) |
| Multiple Sclerosis | 1767 (0.4%) | 842 (0.4%) |
| Parkinson’s | 847 (0.2%) | 359 (0.2%) |
| Peptic Ulcer Disease | 6046 (1.2%) | 4894 (2.2%) |
| Peripheral Vascular Disease | 1415 (0.3%) | 1219 (0.6%) |
| Prostate Disorder | 8253 (1.7%) | 6570 (3.0%) |
| Substance Misuse | 97 (0.02%) | 668 (0.3%) |
| Connective Tissue Disease | 7343 (1.5%) | 4741 (2.2%) |
| Schizophrenia or Bipolar | 1970 (0.4%) | 3707 (1.7%) |
| Stroke or TIA | 8197 (1.6%) | 3472 (1.6%) |
| Thyroid Disorders | 28487 (5.7%) | 13848 (6.3%) |
| No Conditions | 214229 (45.8%) | 77443 (38.0%) |


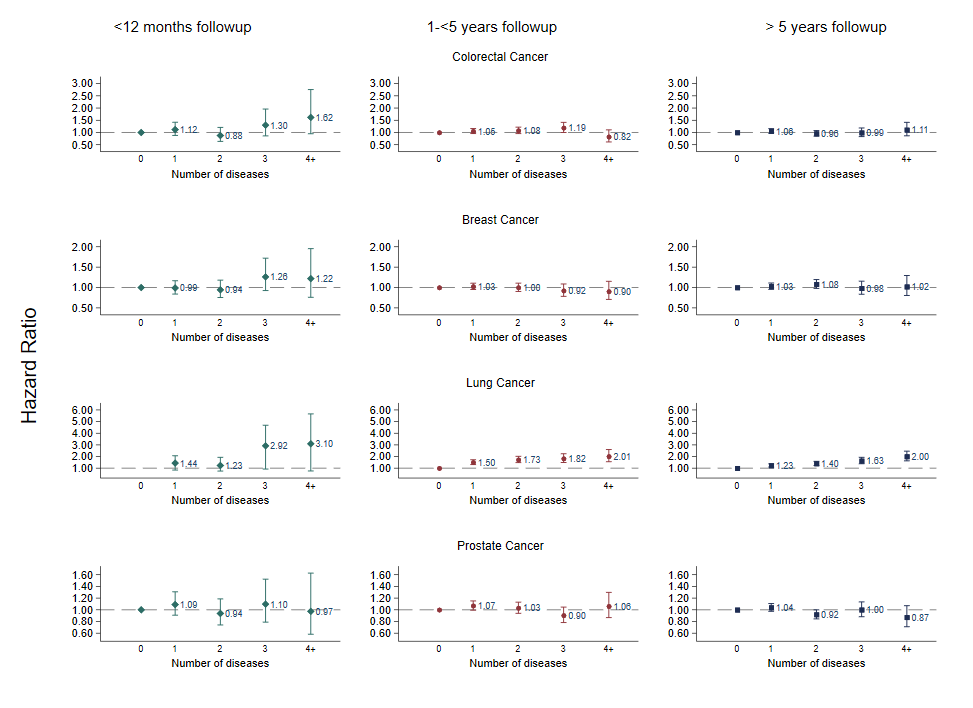


**Supplementary Figure 1: Association between disease count and risk of cancer diagnosis stratified by follow up time in UK Biobank.**

Diamond: less than 12 months follow up; circle: 1-<5 years follow up; square: ≥5 years follow up. All analyses adjusted for sex, region, year of recruitment, ethnicity, year of birth, townsend score, BMI, smoking, alcohol consumption and MHT use (except prostate cancer). Age was used as the underlying time variable.

**Supplementary Table 2: Association between self-reported derived multi-morbidity measures and risk of colorectal and prostate cancer diagnosis by follow-up time in UK Biobank**

Adjusted for age, sex, socioeconomic status, ethnicity, region, year of recruitment, BMI, alcohol consumption, smoking and MHT use.

HR: Hazard ratio. CI: Confidence interval. MM: Multi-morbidity

|  |  | Colorectal cancer | | | | Prostate cancer | | | |
| --- | --- | --- | --- | --- | --- | --- | --- | --- | --- |
|  |  | <1 year follow up | 1-4 years follow up | ≥5 years follow up |  | <1 year follow up | 1-4 years follow up | ≥5 years follow up |  |
|  |  | HR (95% CI) | HR (95% CI) | HR (95% CI) | P for heterogeneity^1^ | HR (95% CI) | HR (95% CI) | HR (95% CI) | P for heterogeneity^1^ |
| Weighted Cambridge MM Score Quartiles | 0 | Reference | Reference | Reference | 0.99 | Reference | Reference | Reference | 0.73 |
|  | 1 | 1.10 (0.84 - 1.46) | 1.05 (0.94 - 1.18) | 1.06 (0.96 - 1.18) |  | 1.18 (0.96 - 1.45) | 1.11 (1.02 - 1.21) | 1.07 (1.00 - 1.16) |  |
|  | 2 | 1.04 (0.71 - 1.53) | 1.03 (0.88 - 1.20 | 1.01 (0.87 - 1.16) |  | 1.24 0.94 - 1.63) | 1.09 (0.97 - 1.22) | 1.06 (0.96 - 1.17) |  |
|  | 3 | 0.99 (0.71 - 1.37) | 1.02 (0.90 - 1.16) | 0.91 (0.80 - 1.03) |  | 0.99 (0.78 - 1.28) | 1.05 (0.95 - 1.16) | 0.98 (0.90 - 1.07) |  |
|  | 4 | 1.18 (0.87 - 1.58) | 1.15 (1.02 - 1.30) | 1.11 (0.99 - 1.25) |  | 0.81 (0.63 - 1.03) | 0.94 (0.85 - 1.03) | 0.87 (0.80 - 0.95) |  |
|  | Trend | 1.02 (0.95 – 1.10) p=0.47 | 1.02 (1.00 – 1.06) p=0.07 | 1.01 (0.98 – 1.03) p=0.57 |  | 0.95 (0.91 – 1.01) p=0.11 | 0.98 (0.97 – 1.01) p=0.32 | 0.97 (0.96 – 0.99) p=0.005 |  |
| Number of diseases | 0 | Reference | Reference | Reference | 0.16 | Reference | Reference | Reference | 0.45 |
|  | 1 | 1.12 (0.88 - 1.42) | 1.05 (0.96 - 1.16) | 1.06 (0.97 - 1.16) |  | 1.09 (0.91 - 1.31) | 1.07 (0.99 - 1.15) | 1.04 (0.97 - 1.11) |  |
|  | 2 | 0.88 (0.64 - 1.21) | 1.08 (0.95 - 1.22) | 0.96 (0.85 - 1.08) |  | 0.94 (0.74 - 1.19) | 1.03 (0.94 - 1.13) | 0.92 (0.84 - 1.00) |  |
|  | 3 | 1.30 (0.86 - 1.96) | 1.19 (1.00 - 1.41) | 0.99 (0.83 - 1.18) |  | 1.10 (0.79 - 1.52) | 0.90 (0.78 - 1.05) | 1.00 (0.88 - 1.14) |  |
|  | ≥4 | 1.62 (0.95 - 2.76) | 0.82 (0.61 - 1.11) | 1.11 (0.86 - 1.42) |  | 0.97 (0.58 - 1.63) | 1.06 (0.87 - 1.30) | 0.87 (0.71 - 1.07) |  |
|  | Trend | 1.06 (0.96 – 1.17) p=0.25 | 1.02 (0.98 – 1.07) p=0.30 | 1.00 (0.96 – 1.04) p=0.93 |  | 0.99 (0.92 – 1.08) p=0.96 | 1.00 (0.97 – 1.03) p=1.00 | 0.97 (0.95 - 1.01) p=0.14 |  |
| Multimorbidity | No | Reference | Reference | Reference | 0.52 | Reference | Reference | Reference | 0.49 |
|  | Yes | 0.99 (0.78 - 1.25) p=0.91 | 1.05 (0.95 - 1.16) p=0.31 | 0.95 (0.87 - 1.05) p=0.31 |  | 0.93 (0.78 - 1.12) p=0.47 | 0.97 (0.90 - 1.04) p =0.41 | 0.92 (0.86 - 0.98) p=0.01 |  |

1: test restricted to 1-<5 years and ≥5 years strata only

**Supplementary Table 3: Association between self-reported derived multi-morbidity measures and risk of breast and lung cancer diagnosis by follow-up time in UK Biobank**

Adjusted for age, sex, socioeconomic status, ethnicity, region, year of recruitment, BMI, alcohol consumption, smoking and MHT use.

HR: Hazard ratio. CI: Confidence interval. MM: Multi-morbidity

|  | | Breast Cancer | | |  | Lung Cancer | | |  |
| --- | --- | --- | --- | --- | --- | --- | --- | --- | --- |
|  |  | <1 year follow up | 1-4 years follow up | ≥5 years follow up |  | <1 years follow up | 1-4 years follow up | ≥5 years follow up |  |
|  |  | HR (95% CI) | HR (95% CI) | HR (95% CI) | P for heterogeneity^1^ | HR (95% CI) | HR (95% CI) | HR (95% CI) | P for heterogeneity^1^ |
| Weighted Cambridge MM Score Quartiles | 0 | Reference | Reference | Reference | 0.95 | Reference | Reference | Reference | 0.33 |
|  | 1 | 1.04 (0.86 - 1.27) | 1.04 (0.95 - 1.14) | 1.06 (0.96 - 1.16) |  | 1.16 (0.74 - 1.82) | 1.42 (1.20 - 1.66) | 1.15 (1.00 - 1.31) |  |
|  | 2 | 0.90 (0.69 - 1.16) | 1.01 (0.90 - 1.12) | 1.02 (0.92 - 1.14) |  | 1.23 (0.69 - 2.19) | 1.34 (1.08 - 1.65) | 1.06 (0.88 - 1.26) |  |
|  | 3 | 1.04 (0.84 - 1.29) | 0.98 (0.88 - 1.08) | 1.02 (0.92 - 1.12) |  | 1.25 (0.78 - 2.01) | 1.59 (1.34 - 1.87) | 1.28 (1.11 - 1.46) |  |
|  | 4 | 1.01 (0.79 - 1.28) | 1.00 (0.89 - 1.11) | 1.06 (0.95 - 1.18) |  | 2.56 (1.76 - 3.73) | 2.01 (1.73 - 2.33) | 1.79 (1.59 - 2.02) |  |
|  | Trend | 1.00 (0.95 – 1.05) p=0.94 | 0.99 (0.97 – 1.02) p=0.71 | 1.01 (0.99 – 1.03) p=0.40 |  | 1.24 (1.14 – 1.37) p<0.001 | 1.17 (1.13 – 1.21) p<0.001 | 1.14 (1.11 – 1.17) p<0.001 |  |
| Number of diseases | 0 | Reference | Reference | Reference | 0.59 | Reference | Reference | Reference | 0.08 |
|  | 1 | 0.99 (0.84 - 1.17) | 1.03 (0.95 - 1.11) | 1.03 (0.95 - 1.11) |  | 1.44 (1.00 - 2.07) | 1.50 (1.31 - 1.72) | 1.23 (1.10 - 1.37) |  |
|  | 2 | 0.94 (0.75 - 1.18) | 1.00 (0.90 - 1.11) | 1.08 (0.98 - 1.19) |  | 1.23 (0.79 - 1.93) | 1.73 (1.48 - 2.02) | 1.40 (1.23 - 1.59) |  |
|  | 3 | 1.26 (0.92 - 1.72) | 0.92 (0.78 - 1.09) | 0.98 (0.83 - 1.15) |  | 2.92 (1.82 - 4.69) | 1.82 (1.49 - 2.24) | 1.63 (1.38 - 1.93) |  |
|  | ≥4 | 1.22 (0.76 - 1.95) | 0.90 (0.71 - 1.15) | 1.02 (0.80 - 1.29) |  | 3.10 (1.69 - 5.66) | 2.01 (1.55 - 2.60) | 2.00 (1.63 - 2.46) |  |
|  | Trend | 1.04 (0.96 – 1.12) p=0.36 | 0.99 (0.95 – 1.02) p=0.47 | 1.02 (0.98 – 1.05) p=0.35 |  | 1.33 (1.18 – 1.51) p<0.001 | 1.20 (1.14 – 1.26) p<0.001 | 1.18 (1.13 – 1.23) p<0.001 |  |
| Multimorbidity | No | Reference | Reference | Reference | 0.36 | Reference | Reference | Reference | 0.95 |
|  | Yes | 1.04 (0.88 - 1.25) p=0.62 | 0.96 (0.88 - 1.05) p=0.35 | 1.04 (0.96 -1.13) p=0.36 |  | 1.50 (1.11 - 2.03) p=0.01 | 1.42 (1.27 - 1.60) p<0.001 | 1.37 (1.24 - 1.50) p<0.001 |  |

1: test restricted to 1-<5 years and ≥5 years strata only

**Supplementary table 4: Association between multi-morbidity measures and risk of diagnosis of colorectal, breast, lung and prostate cancer**

Adjusted for age, sex, socioeconomic status, ethnicity, region, year of recruitment, BMI, smoking status, alcohol consumption and MHT use (not prostate cancer).

MM: Multi-morbidity . CI: Confidence Interval

|  | | Colorectal Cancer | Breast Cancer | Lung Cancer | Prostate Cancer |
| --- | --- | --- | --- | --- | --- |
|  |  | HR (95% CI) | HR (95% CI) | HR (95% CI) | HR (95% CI) |
| Weighted Cambridge MM Score Quartiles | 0 | Reference | Reference | Reference | Reference |
|  | 1 | 1.06 (0.98 - 1.14) | 1.05 (0.98 - 1.12) | 1.24 (1.12 - 1.38 ) | 1.09 (1.03 - 1.15) |
|  | 2 | 1.02 (0.92 - 1.13) | 1.02 (0.94 - 1.10) | 1.16 (1.01 - 1.33) | 1.07 (0.99 - 1.16) |
|  | 3 | 0.96 (0.88 - 1.05) | 0.99 (0.93 - 1.07) | 1.39 (1.25 - 1.54) | 1.01 (0.94 - 1.08) |
|  | 4 | 1.13 (1.04 - 1.23) | 1.03 (0.95 - 1.11) | 1.87 (1.70 - 2.05) | 0.90 (0.84 - 0.96) |
|  | P for trend | 0.22 | 0.09 | <0.001 | 0.11 |
| Number of diseases | 0 | Reference | Reference | Reference | Reference |
|  | 1 | 1.06 (0.99 - 1.13) | 1.03 (0.98 - 1.09) | 1.33 (1.22 - 1.44) | 1.05 (1.00 - 1.10) |
|  | 2 | 1.01 (0.93 - 1.10) | 1.04 (0.97 - 1.12) | 1.52 (1.37 - 1.67) | 0.97 (0.91 - 1.03) |
|  | 3 | 1.08 (0.96 - 1.23) | 0.95 (0.85 - 1.07) | 1.70 (1.49 - 1.94) | 0.95 (0.87 - 1.05) |
|  | ≥4 | 0.97 (0.80 - 1.17) | 0.97 (0.82 - 1.15) | 2.00 (1.70 - 2.35) | 0.96 (0.83 - 1.11) |
|  | P for trend | 0.42 | 0.60 | <0.001 | 0.30 |
| MM | No | Reference | Reference | Reference | Reference |
|  | Yes | 1.00 (0.93 - 1.07) | 1.00 (0.95 - 1.06) | 1.39 (1.29 - 1.49) | 0.94 (0.89 - 0.99) |
|  | P for trend | 0.95 | 0.94 | <0.001 | 0.01 |

**Supplementary Table 5: LRχ^2^ values for minimally and fully adjusted models for each cancer**

Min – adjusted for age, sex (UKB only), socioeconomic status, ethnicity, region, year of recruitment. Max – adjusted as for min plus BMI, smoking status, alcohol consumption and MHT use

|  | Colorectal Cancer | | | Breast Cancer | | | Lung Cancer | | | Prostate Cancer | | |
| --- | --- | --- | --- | --- | --- | --- | --- | --- | --- | --- | --- | --- |
|  | Min | Max | Change | Min | Max | Change | Min | Max | Change | Min | Max | Change |
|  | LRχ^2^ | LRχ^2^ | % | LRχ^2^ | LRχ^2^ | % | LRχ^2^ | LRχ^2^ | % | LRχ^2^ | LRχ^2^ | % |
| Weighted Cambridge MM Score Quartiles | 19.3 | 12.6 | 34.5% | 5.4 | 2.7 | 52.0% | 317.2 | 177.5 | 44.0% | 44.8 | 33.1 | 53.0% |
| Number of diseases | 9.9 | 6.2 | 37.2% | 5.7 | 3.5 | 37.0% | 203.8 | 128.8 | 36.8% | 16.0 | 10.3 | 35.6% |
| Multimorbidity | 0.4 | 0.2 | 59.5% | 0.9 | 0.12 | 99.3% | 132.6 | 75.6 | 42.9% | 12.9 | 6.1 | 26.0% |

**Supplementary Table 6: Distribution of covariates by number of diseases in the UK Biobank subset with primary care data, restricted to those with at least 12 months follow up**

| Number of diseases | Overall | Age median(IQR) | Sex | Ethnicity | SES  median(IQR) | BMI  median(IQR) | Smoking | Alcohol | MHT |
| --- | --- | --- | --- | --- | --- | --- | --- | --- | --- |
|  |  |  | Female (N(%)) | White (N(%)) |  |  | Current (N(%)) | Daily | Ever (N(%)) |
| 0 | 77443  (38.0%) | 55 (48 - 61) | 39188 (36.0%) | 73218 (37.9%) | -2.25 (-3.7 - 0.2) | 26.3 (23.8 - 29.1) | 7637 (35.8%) | 15760 (39.2%) | 11515 (28.1%) |
| 1 | 58718  (28.8%) | 58 (50 - 63) | 31614 (29.0%) | 55700 (28.8%) | -2.24 (-3.7 - 0.2) | 26.8 (24.2 - 29.8) | 5805 (27.2%) | 11956 (29.7%) | 11733 (28.6%) |
| 2 | 34920  (17.1%) | 59 (52 - 64) | 19406 (17.8%) | 33109 (17.1%) | -2.13 (-3.6 - 0.6) | 27.2 (24.5 - 30.5) | 3730 (17.5%) | 6803 (16.9%) | 8210 (20.0%) |
| 3 | 17963  (8.8%) | 60 (53 - 64) | 10150 (9.3%) | 17051 (8.8%) | -1.93 (-3.5 - 0.9) | 27.8 (24.9 - 31.3) | 2165 (10.1%) | 3324 (8.3%) | 4872 (11.9%) |
| 4+ | 14872  (7.3%) | 61 (55 - 65) | 8539 (7.8%) | 14110 (7.3%) | -1.49 (-3.3 - 1.7) | 28.7 (25.5 - 32.7) | 2021 (9.5%) | 2394 (5.9%) | 4628 (11.3%) |

**Supplementary Table 7: Association between self-reported individual diseases at recruitment and risk of diagnosis of lung cancer**

HR: Hazard ratio. CI: Confidence interval. Bold p-value indicate association maintains statistical significance following correction for multiple testing

| Disease | Overall |  | Fully Adjusted | | |
| --- | --- | --- | --- | --- | --- |
|  | N (%) | N (%) | HR | 95% CI | p-value |
| Alcohol Problems | 810 (0.2%) | 33 (0.9%) | 2.06 | (1.43 - 2.95) | **<0.001** |
| Anorexia/ Bulimia | 370 (0.1%) | 0 (0%) |  |  |  |
| Anxiety | 11806 (2.3%) | 80 (2.2%) | 0.89 | (0.71 - 1.13) | 0.34 |
| Asthma | 58257 (11.6%) | 459 (12.8%) | 1.21 | (1.09 - 1.34) | **<0.001** |
| Atrial Fibrillation | 3714 (0.7%) | 37 (1.0%) | 1.04 | (0.74 - 1.47) | 0.81 |
| Bronchiectasis | 4761 (0.9%) | 43 (1.2%) | 0.99 | (0.72 - 1.37) | 0.97 |
| Chronic Kidney Disease | 232 (0.0%) | 4 (0.1%) | 1.79 | (0.58 - 5.55) | 0.32 |
| Chronic Liver disease | 2076 (0.4%) | 25 (0.7%) | 1.29 | (0.86 - 1.93) | 0.22 |
| Chronic Sinusitis | 3099 (0.6%) | 19 (0.5%) | 0.83 | (0.51 - 1.33) | 0.44 |
| Constipation | 402 (0.1%) | 5 (0.1%) | 2.13 | (0.89 - 5.14) | 0.09 |
| COPD | 11803 (2.3%) | 409 (11.4%) | 2.45 | (2.20 - 2.73) | **<0.001** |
| Coronary artery disease | 22930 (4.6%) | 449 (12.5%) | 1.52 | (1.37 - 1.69) | **<0.001** |
| Dementia | 297 (0.1%) | 2 (0.1%) | 0.46 | (0.07 - 3.28) | 0.44 |
| Depression | 28446 (5.7%) | 272 (7.6%) | 1.11 | (0.98 - 1.26) | 0.11 |
| Diabetes | 25712 (5.1%) | 311 (8.7%) | 1.16 | (1.03 - 1.32) | **0.02** |
| Diverticular disease | 5391 (1.1%) | 65 (1.8%) | 1.15 | (0.89 - 1.48) | 0.28 |
| Psoriasis or eczema | 18491 (3.7%) | 146 (4.1%) | 1.10 | (0.93 - 1.31) | 0.26 |
| Epilepsy | 4053 (0.8%) | 47 (1.3%) | 1.46 | (1.08 - 1.96) | **0.01** |
| Heart Failure | 314 (0.1%) | 7 (0.2%) | 1.80 | (0.81 - 4.02) | 0.15 |
| Hypertension | 133206 (26.5%) | 1343 (37.4%) | 1.18 | (1.10 - 1.27) | **<0.001** |
| Inflammatory Bowel Disease | 4229 (0.8%) | 44 (1.2%) | 1.48 | (1.09 - 2.00) | **0.01** |
| Irritable bowel syndrome | 11481 (2.3%) | 80 (2.2%) | 0.93 | (0.74 - 1.17) | 0.53 |
| Migraine | 14380 (2.9%) | 85 (2.4%) | 1.05 | (0.84 - 1.32) | 0.65 |
| Multiple Sclerosis | 1777 (0.4%) | 13 (0.4%) | 0.98 | (0.57 - 1.69) | 0.94 |
| Parkinson’s | 855 (0.2%) | 6 (0.2%) | 1.00 | (0.45 - 2.24) | 0.99 |
| Peptic Ulcer Disease | 6122 (1.2%) | 113 (3.1%) | 1.38 | (1.14 - 1.68) | **0.001** |
| Peripheral Vascular Disease | 1434 (0.3%) | 30 (0.8%) | 2.20 | (1.50 - 3.21) | **<0.001** |
| Prostate Disorder | 8389 (1.7%) | 90 (2.5%) | 0.96 | (0.77 - 1.20) | 0.74 |
| Substance Misuse | 98 (0.0%) | 6 (0.2%) | 3.63 | (1.50 - 8.76) | **0.004** |
| Connective Tissue Disease | 7431 (1.5%) | 89 (2.5%) | 1.15 | (0.92 - 1.43) | 0.22 |
| Schizophrenia or Bipolar | 1995 (0.4%) | 24 (0.7%) | 1.11 | (0.73 - 1.67) | 0.63 |
| Stroke or TIA | 8312 (1.7%) | 148 (4.1%) | 1.34 | (1.13 - 1.59) | **0.001** |
| Thyroid Disorders | 28700 (5.7%) | 200 (5.6%) | 0.87 | (0.75 - 1.02) | 0.08 |

Adjusted for sex, region, year of recruitment, ethnicity year of birth, townsend score, BMI, smoking, alcohol consumption and MHT use. Age was used as the underlying time variable.
